# Spatiotemporal Distribution of Tuberculosis during Urbanization in the New Urban Area of Nanchang City, China, 2010–2018

**DOI:** 10.3390/ijerph16224395

**Published:** 2019-11-11

**Authors:** Shu Yang, Yuan Gao, Wei Luo, Longfu Liu, Yuanhua Lei, Xiaoling Zhang

**Affiliations:** 1 The Collaboration Unit for Field Epidemiology of State Key Laboratory, for Infectious Disease Prevention and Control, Jiangxi Provincial key Laboratory of Animal-origin and Vector-borne Diseases, Nanchang Center for Disease Control and Prevention, Nanchang 330038, China; younsoo@163.com (S.Y.); 8127@sina.com (W.L.); 2 State Key Laboratory of Infectious Disease Prevention and Control, National Institute for Communicable Disease Control and Prevention, Chinese Center for Disease Control and Prevention, Beijing 102206, China; gaoyuancdc@126.com; 3 XinJian Center for Disease Control and Prevention, Nanchang 330100, China; ysnccdc@163.com (L.L.); xjxjfkx@163.com (Y.L.)

**Keywords:** tuberculosis, spatiotemporal distribution, urbanization

## Abstract

Background: Urbanization will play a key role in ending the tuberculosis (TB) epidemic by 2030, but understanding the relationship between urbanization and the health threats posed by TB is incomplete. Therefore, this study aimed to explore the spatiotemporal distribution of TB at the township level during urbanization in the new urban area of Nanchang. Methods: Seasonal-trend decomposition of time series analysis was used to explore the seasonal distribution and trend analysis. Global and local spatial autocorrelation statistics, and space–time scan statistics were performed to detect the spatiotemporal clusters of TB cases in the new urban area of Nanchang from 2010 to 2018. Results: A total of 3245 TB cases were reported in the study area from 2010 to 2018. Of all the TB cases, 68% occurred in individuals older than 40 years old, 73.2% were male cases, and 56.6% were farmers. The primary seasonal peak was in late spring (April), and a smaller peak was in early autumn (September). The results of local indicators of spatial association showed that Jiaoqiao town and Changleng town might be “High–High” clusters. The most likely spatiotemporal cluster was located in the southwest of the study area in 2010, which included five towns, and then shifted to the northeast gradually. Across 2010 to 2018, nine spatiotemporal clusters were identified. The most likely cluster was located at the northeast of the study area. The center of this area was in Nanji town with a circle radius of 43.74 kilometers. Conclusions: The spatial clusters of TB incidence shifted to the rural region and the fringe of the new urban area of Nanchang. Targeted management strategies for urban migrants in the process of urbanization should be strengthened.

## 1. Introduction

Tuberculosis (TB), a major cause of morbidity and premature mortality, is an infectious disease caused by mycobacterium TB, and mainly transmits through the respiratory tract. It is characterized by a high frequency of latent TB infection (LTBI), which tends to reactivate years or decades after the initial infection [1]. This means that TB can easily spread among the population if a one smear-positive case occurs, then followed by an outbreak. Although concerted efforts to confine the prevalence of TB worldwide have been undertaken, it remains a major public health issue with a high global health burden because of the low detection, high incidence, multiple drug resistance, and co-infection [2]. It was estimated that about 10.0 million people around the world suffered from TB infection in 2017 [3]. China has the second largest burden of TB worldwide. In 2018, TB was the second cause of morbidity and mortality among all of the notifiable infectious diseases, according to the national report of notifiable infectious disease [4]. Nanchang, the capital of Jiangxi Province, is located in southeastern China, and has a high TB burden [5]. The annual TB surveillance report of Nanchang from 2010 to 2018 showed that the new urban area of Nanchang had the highest TB incidence in Nanchang.

The new urban area and urban–rural integration has experienced an urbanization process at an unprecedented pace over the past 15 years, with rapid environmental improvements and population movements [6]. It has been reported in [7,8] that urbanization could increase the risk of TB infection and transmission through the following ways. First, spatial clustering of TB is closely associated with a migrant population. For example, population movements increased TB incidence in Beijing [9]. Second, there are more rural migrants with a high risk of TB infection, who have a lower social and economic level and a limited knowledge of TB prevention, moving to urban areas [10]. In addition, as an infectious disease transmitted via the respiratory tract, TB is conducive to outbreaks in urban areas with a high density of population. On the contrary, World Health Organization (WHO) experts [11] suggest that urbanization could provide an opportunity to combat infectious diseases like TB, because more people would enjoy better infrastructure and services including housing and medical care that would help combat poverty and inequality. 

Therefore, the current understanding of the relationship between urbanization and the health threats posed by TB is incomplete. More evidence should be gathered to increase our understanding of the spatiotemporal dynamics of TB after urbanization. In recent years, spatiotemporal analysis has been used frequently to characterize spatial epidemiology of diseases because of the power of quantitative statistics and mapping visualization [12,13,14,15,16,17]. As a communicable disease, the distribution of TB has spatial heterogeneity and spatial scale dependence. Therefore, it is important to understand the distribution and aggregation degree of TB cases through different spatial scales. Presently, some studies [14,18,19,20] have explored the spatiotemporal distribution of TB at a large scale level such as at the provincial and the prefecture level. However, this does not take full advantage of the spatial information of TB cases, which means that some smaller aggregation units are masked. In addition, TB tends to spread locally, once a TB case of LTBI has not been found and followed up in a timely manner. Therefore, it is necessary to understand the spatiotemporal patterns of TB at a smaller scale in order to formulate precise control measures in local areas. However, few studies have explored the spatiotemporal distribution at the township or street level in China, especially in Jiangxi. This study aimed to explore the spatiotemporal distribution of TB in the new urban area of Nanchang at the township level, to understand the spatial variation of TB after rapid urbanization, and to provide more scientific information for ending the TB epidemic by 2030 [11]. 

## 2. Materials and Methods 

### 2.1. Study Area

**Figure 1:**
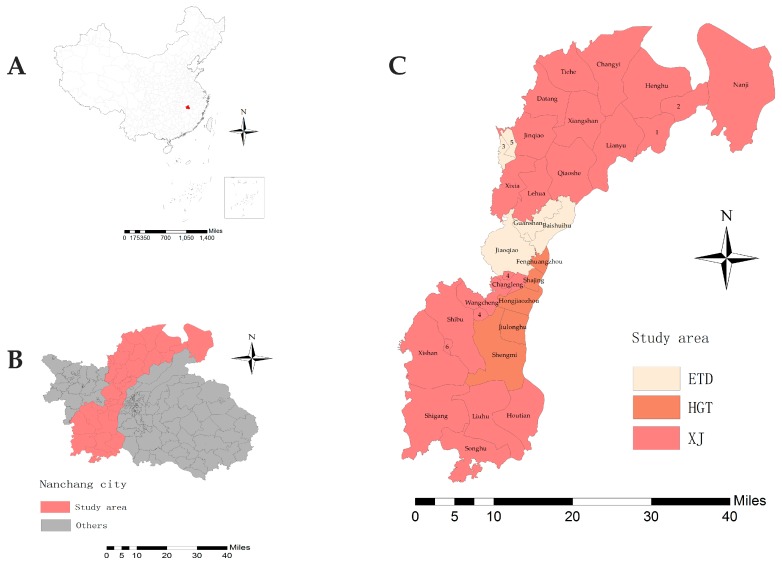
The location of the study area. (**A**) Location of Nanchang city in China. (**B**) Location of the new urban area of Nanchang city. (**C**) Administrative division of the study area (1. Chengxin Industrial Co. Ltd.; 2. Zhugang Industrial Co. Ltd.; 3. Jiangxi Sanghai Group Co. Ltd.; 4. Foreign Invested Industrial Zone of Jiangxi Changleng; 5. Xinqizhou; 6. Xinfeng Farm). The maps were created in ArcGIS software (version 10.2, ESRI Inc., Redlands, CA, USA).

The new urban area of Nanchang is a rural–urban continuum with a population of 1,180,000, and an area of 2597.32 km^2^. It includes one county and two districts, Xinjian county (XJ), Nanchang Economic and Technological Development Zone (ETD), and Honggutan new district (HGT) (Figure 1). ETD was independent from XJ between 1987 and 1993 due to the urbanization process, and HGT between 2004 and 2009 [6]. In recent years, the speed of urbanization in the new urban area is the fastest in the whole province, and HGT has become the political, cultural, and economic center of Jiangxi Province [21]. XJ has been a municipal district since 2015, and transferred from an agriculture-oriented development area to an urbanization and industrialization area [22]. At present, urbanization is taking place in the southwest of the new urban area. 

### 2.2. Data Collection

TB is reported as a class B notifiable communicable disease, which should be reported to the Chinese Center for Disease Control and Prevention (China CDC) within 24 hours after the diagnosis. The case report work of the whole new urban area belongs to the centralized management of the XinJian Center for Disease Control and Prevention. Daily TB data of the study area from 2010 to 2018 were obtained from the China Information System for Disease Control and Prevention from China CDC. Clinically diagnosed and laboratory based diagnosed cases were included in our study. Demographic information and diagnosis information were included. For example, information on gender, age, occupation, reporting date, residential address, and case classification were included. 

The annual population data for each administrative area were obtained from the Nanchang Statistical Yearbook. The resident population, who had lived in the new urban area for more than six months, were included in our study.

A township-level based map was acquired from the data center for geographic sciences and natural sources research, Chinese Academy of Sciences (CAS) for the spatial analysis [23].

### 2.3. Data Analysis

A descriptive analysis was utilized to determine the epidemiological characteristics in IBM SPSS Statistics for Windows (version 20.0; IBM Corp., Armonk, NY, USA). 

A seasonal-trend decomposition of the time series analysis was conducted to explore the seasonal distribution, and trend analysis in R 3.1.1 (AT&T BellLaboratories, Auckland, New Zealand,) [24]. Global spatial autocorrelation analysis and local indicators of spatial association (LISA) were performed in ArcGIS (v10.4.1, ESRI Inc., Redlands, CA, USA) to visualize the global and local spatial clustering patterns of TB cases during 2010 to 2018 [14,15]. Global Moran’s I Index, which ranged from −1 to 1, was used to analyze global spatial autocorrelation. Moran’s Index = 0 suggested a random spatial distribution. Moran’s Index < 0 suggested a dispersing in the spatial distribution, and Moran’s I > 0 suggested clustering in the spatial distribution. LISA was used to explore significant hot spots (High–High), cold spots (Low–Low), and spatial outliers (High–Low and Low–High) by calculating the local Moran’s I between a given location and the average of neighboring values in the surrounding locations. A Kulldorff’s space–time scan statistical analysis was used to detect the spatial–temporal clusters of TB in the study area in SaTScan software (version 9.4.4) [25,26]. The discrete Poisson probability model by a circular window with a radius was used for scanning. The maximum spatial cluster size and maximum temporal cluster size were all set to 15% [19,27,28]. A *p* value of less than 0.05 was considered to be statistically significant.

The maps were created in ArcGIS software (version 10.2, ESRI Inc.; Redlands, CA, USA).

## 3. Results

### 3.1. Descriptive Statistics

A total of 3245 TB cases were reported in the new urban area from 2010 to 2018 including 1429 (44.0%) clinically diagnosed cases, and 1816 (56.0%) confirmed cases. Male cases were 2375 (73.2%), and the male-to-female ratio (not significant) ranged from 3.81:1 in 2010 to 2.40:1 in 2016. A total of 68% of the TB cases occurred in individuals greater than 40 years old, and different age group ratios of different years were significantly different (*χ*^2^ = 72.366, *p* = 0.000). The majority of the TB cases were concentrated in farmers (56.6%), followed by students (10.2%). Different occupational ratios of different years were significantly different (*χ*^2^ = 387.224, *p* = 0.000). Additionally, sputum smear-positive (SS+) TB cases increased first and then decreased, and were higher than other types of diagnosis cases (Table 1). 

**Figure 2:**
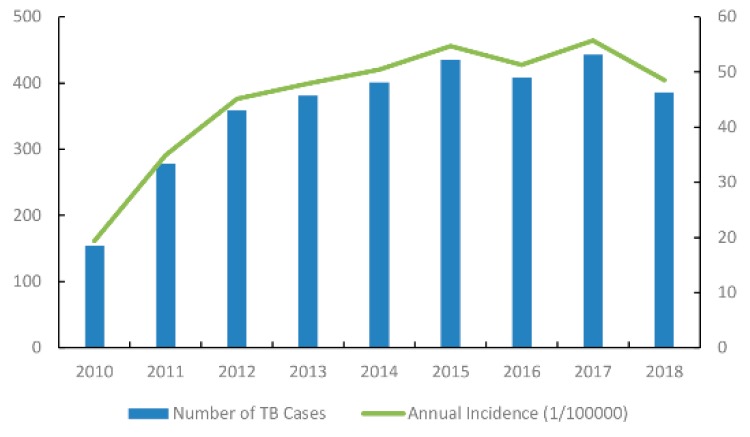
Annual tuberculosis case numbers and incidences in the new urban area of Nanchang from 2010 to 2018.

**Figure 3:**
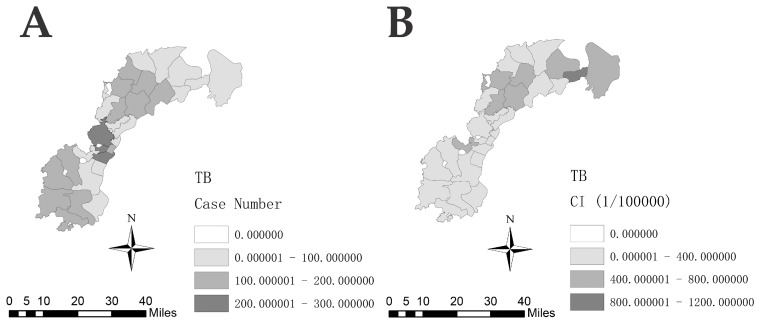
Spatial distribution of tuberculosis cases in the new urban area of Nanchang, 2010–2018. (**A**) Spatial distribution of TB cases. (**B**) Cumulative incidence of TB cases.

The annual case numbers ranged from 154 in 2010 to 443 in 2017, and presented a totally stable trend from 2013 to 2018 (Figure 2). The cumulative number of TB cases in each town ranged from 0 to 284, among which Jiaoqiao town had the highest (284 cases). The cumulative incidence of each town ranged from 0 to 1140 per 100,000 population, and the highest TB cumulative incidence occurred in Zhugang Industrial Co. Ltd (1140 per 100,000 population) (Figure 3).

### 3.2. Seasonal Decomposition Analyses 

**Figure 4:**
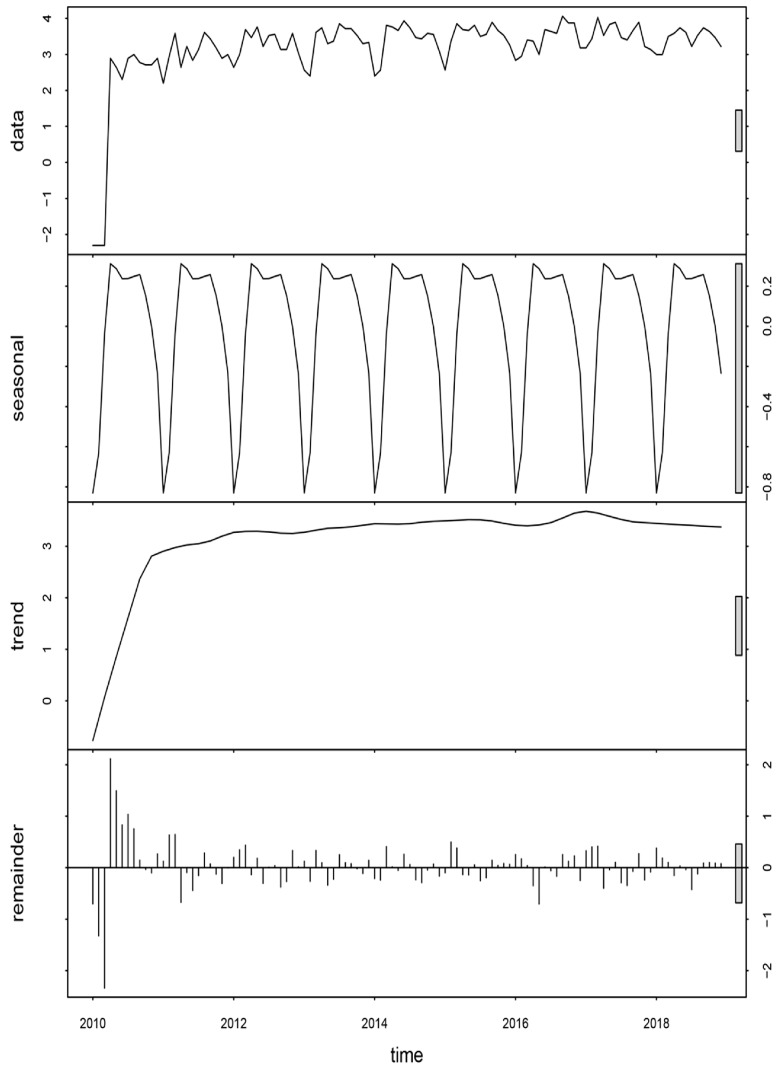
Decomposed tuberculosis cases in the new urban area of Nanchang from 2010 to 2018.

A seasonal-trend decomposition of a time series analysis was performed to capture the long-term changes, the periodic changes over the course of a year, and the changes that could not be explained by seasonal and trend components (Figure 4). There was a rhythmic fluctuation in the raw data. After eliminating part of the random noise or reminder component, the raw data showed an obvious seasonal pattern. There were two seasonal peaks: the primary peak was in late spring (April), and a smaller peak was in early autumn (September). In addition, the number of TB cases also showed a steady trend with the interannual pattern presenting a small peak in 2017.

### 3.3. Spatiotemporal Analysis

#### 3.3.1. Spatial Autocorrelation Analysis

The results of global spatial autocorrelation showed that the annual global Moran’s *I* indexes, which ranged from –0.016 to 0.098, failed to pass the significance level test (*p* < 0.05), suggesting that there was no global spatial autocorrelation, and the distribution of TB was random from 2010 to 2018.

**Figure 5:**
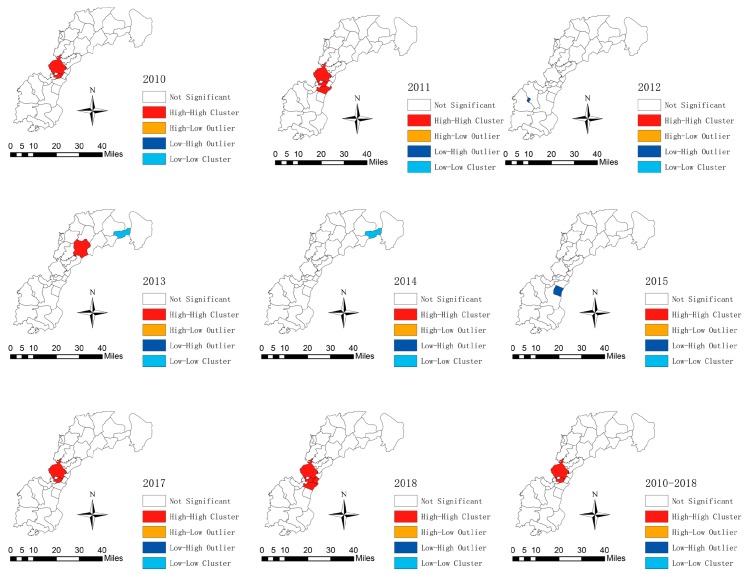
Local indicators of spatial association cluster maps for tuberculosis in the new urban area of Nanchang from 2005 to 2017.

The LISA cluster map showed that Jiaoqiao and Changleng towns were identified as the hot spots (High–High cluster area) in the 8-years duration. The hot spots disappeared in 2012, 2014, 2015, and moved to Qiaoshe town in 2013, but the main hot spots were around Jiaoqiao township. Chengxin Industrial Co. Ltd. was a cold spot (Low–Low cluster area) in 2013 and 2014 (Figure 5).

#### 3.3.2. Spatiotemporal Cluster Analysis 

**Figure 6:**
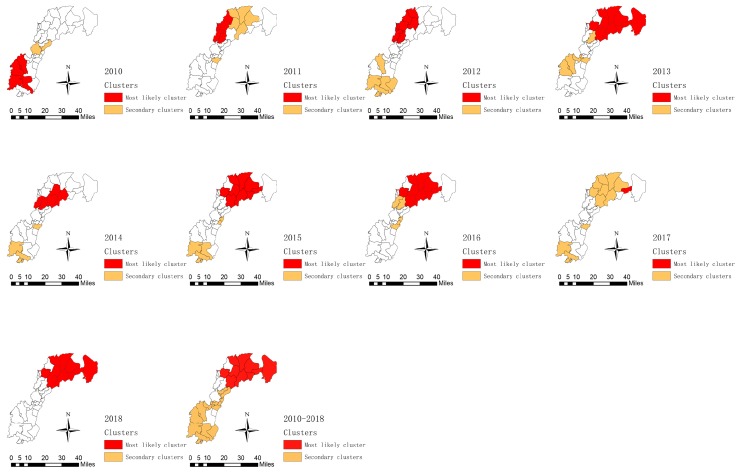
Yearly spatiotemporal clusters of tuberculosis cases in the new urban area of Nanchang from 2010 to 2018 using Kulldorff’s space–time scan statistic.

The incidence of TB was aggregated through space and time using Kulldorff’s space–time scan statistics. The results showed that the most likely cluster changed a lot from 2010 to 2018. This was located in the southwest of the study area in 2010 including five towns, then shifted to the northeast of the study area gradually, and stayed in the northeast from 2015 to 2018 (Figure 6). 

A most likely cluster, and eight secondary clusters were detected during the study period (Figure 6). The most likely cluster was located at the northeast of the study area. The expected case number was 133.91, but the observed case number was 328, where the relative risk for the analysis was 2.61 (LLR = 106.02, *p* = 0.000). The center of this area was in Nanji town, with a circle radius of 43.74 km including 10 towns. A total of 185,214 people were included with a time frame from 3/1/2015 to 10/31/2017 (Table 2). Secondary clusters were located in 17 towns.

## 4. Discussion

In 2015, 193 Member States of the United Nations adopted the target of ending the TB epidemic by 2030, and one study [11] reported that urbanization would play a key role in supporting TB control measures. However, urbanization is usually followed by rapid environmental changes and population movements, which are implicated as drivers of the transmission dynamics of communicable diseases [8]. This study showed that annual case numbers ranged from 154 in 2010 to 443 in 2017, with a higher proportion of sputum smear-positive (SS+) TB cases. It was observed that after rapid urbanization construction in the new urban area of Nanchang, the number of smear-positive TB cases increased. An explanation can be that the transmission of a certain mycobacterium TB strain is self-limiting with a mean distance of approximately 2000 meters among cases, and generally occurs in a larger but delimited area [29]. The spatial distance of TB transmission is often maintained in a social network within local residents. However, urbanization has connected isolated locations through rural-to-urban migration and short-term travel for commerce and recreation [30]. Therefore, the high population movement could break the stable social network of local residents. Once a new mycobacterium TB lineage carried by migrant population enters into the local social network, that particular strain and its related family strains are more likely to propagate and settle in that network than other strains, which increases the risk of a TB outbreak [29].

Consistent with previous studies [31,32,33,34,35], the results of this study found that individuals older than 40 years old, males, and farmers accounted for most of the TB cases. It was generally recognized that TB was more likely to transmit within areas of poverty with poor housing and sanitation conditions [36,37]. This could be explained by the fact that most migrant workers are male, and live in poor-conditioned houses in the urban fringe.

Additionally, this study showed that the primary seasonal peak was in late spring (April), and a smaller peak was in early autumn (September). A possible cause was that there was a decrease in immune competence in spring [38]. It was of note that students contributed to the second rank of TB cases in our study. There were some reports of TB outbreaks in schools with a high TB incidence [39,40,41]. Since 2004, most colleges and universities in Jiangxi Province have moved to the new urban area of Nanchang to accelerate the urbanization process, which increases the risk of TB outbreak. For example, Hongjiaozhou town of HGT and Jiaoqiao town of ETD are the main location of colleges and universities in Jiangxi Province.

The results of global spatial autocorrelation analysis showed that the distribution of TB was random across all townships from 2010 to 2018. Wang suggested that the global model was probably disturbed by the confounding effect including the heterogeneity of objects and drawing the wrong conclusion [42]. Hence, the local model, LISA, was still implemented to detect the spatial heterogeneity, and revealed that the hot spots disappeared first and then appeared, but the main hot spots were located in Jiaoqiao and Changleng towns. This suggests that the urban fringe had a high TB incidence. In addition, spatiotemporal scan analysis explored the location and extent of the aggregation area, and showed that the most likely cluster was mainly located in the rural region of XJ. HGT and part towns of ETD after urbanization were the secondary clusters. This suggests that the risk of TB transmission in the rural region was higher than that in urban area, which coincided with the process of urbanization across the study area. At present, the process of urbanization is taking place in the southwest of the new urban area of Nanchang. ETD and HGT began to urbanize earlier, and had formed the core part of the new urban area of Nanchang. XJ has become a municipal district, and has been in the process of urbanization since 2015. However, most towns are still rural regions. Therefore, it is of note that urbanization contributes to TB control and prevention through continually improving infrastructure, environmental health, health care provision [43], and urban living [44]. However, population movements caused by urbanization would still be a high risk factor of TB outbreak [11,32]. 

While this study provides important knowledge about the spatiotemporal patterns of TB incidence in the new urban area of Nanchang, there are still certain limitations to our study. Other factors such as environmental and climate factors could have contributed to TB contribution. Therefore, further studies should be conducted in the future.

## 5. Conclusions

This study explored the spatiotemporal dynamics of TB during urbanization in the new urban area of Nanchang, at the township level, and provided a further understanding of the correlation between urbanization and the spatiotemporal distribution of TB. We also found that the spatial clusters of TB incidence in the study area shifted to the rural region and the fringe of the new urban area of Nanchang. Urbanization would still decrease the risk of TB incidence in the long run. Moreover, more research on risk factors should be conducted. 

## Figures and Tables

**Table 1:** Characteristics of all 3245 tuberculosis cases in the new urban area of Nanchang, 2010–2018.

Variable	2010	2011	2012	2013	2014	2015	2016	2017	2018	Total
**Gender**										
Male	122(79.2)	197(70.9)	268(74.7)	295(77.4)	288(71.8)	322(74.0)	288(70.6)	318(71.8)	277(71.8)	2375(73.2)
Female	32(20.8)	81(29.1)	91(25.3)	86(22.6)	113(28.2)	113(26.0)	120(29.4)	125(28.2)	109(28.2)	870(26.8)
**Age group**										
0–20	23(14.9)	23(8.3)	27(7.5)	29(7.6)	26(6.5)	38(8.7)	36(8.8)	31(7.0)	32(8.3)	265(8.2)
20–40	59(38.3)	88(31.7)	72(20.1)	82(21.5)	98(24.4)	86(19.8)	87(21.3)	104(23.5)	98(25.4)	774(23.9)
40–60	28(18.2)	74(26.6)	122(34.0)	109(28.6)	117(29.2)	138(31.7)	107(26.2)	119(26.9)	82(21.2)	896(27.6)
>60	44(28.6)	93(33.5)	138(38.4)	161(42.3)	160(39.9)	173(39.8)	178(43.6)	189(42.7)	174(45.1)	1310(40.4)
**Occupation**										
Farmers	48(31.2)	133(47.8)	229(63.8)	244(64.0)	268(66.8)	260(59.8)	237(58.1)	238(53.7)	181(46.9)	1838(56.6)
Students	38(24.7)	35(12.6)	28(7.8)	30(7.9)	36(9.0)	40(9.2)	44(10.8)	39(8.8)	42(10.9)	332(10.2)
Unemployed	11(7.1)	22(7.9)	24(6.7)	33(8.7)	24(6.0)	38(8.7)	30(7.4)	56(12.6)	55(14.2)	293(9.0)
Retired	13(8.4)	21(7.6)	19(5.3)	27(7.1)	23(5.7)	24(5.5)	26(6.4)	23(5.2)	24(6.2)	200(6.2)
Criminals	12(7.8)	0(0.0)	0(0.0)	1(0.3)	1(0.2)	9(2.1)	22(5.4)	29(6.5)	23(6.0)	85(2.6)
Workers	12(7.8)	14(5.0)	6(1.7)	2(0.5)	6(1.5)	7(1.6)	10(2.5)	6(1.4)	3(0.8)	66(2.0)
Others	29(18.8)	50(18.0)	42(11.7)	31(8.1)	18(4.5)	16(3.7)	22(5.4)	20(4.5)	27(7.0)	255(7.9)
Unknown	3(1.9)	3(1.1)	11(3.1)	13(3.4)	25(6.2)	41(9.4)	17(4.2)	32(7.2)	31(8.0)	176(5.4)
**Diagnosis**										
Sputum smear-positive (SS+) TB	27(17.5)	122(43.9)	232(64.6)	235(61.7)	251(62.6)	261(60.0)	245(60.0)	241(54.4)	175(45.3)	1789(55.1)
Sputum smear-negative(SS-)TB	101(65.5)	114(41.0)	44(12.3)	45(11.8)	69(17.2)	82(18.9)	79(19.4)	99(22.3)	118(30.6)	751(23.1)
Sputum smear not done	5(3.2)	1(0.4)	0(0.0)	2(0.5)	2(0.5)	2(0.5)	6(1.6)	20(4.5)	16(4.1)	54(1.7)
**Tuberculosis Type**										
Tuberculosis pleurisy cases	15(9.7)	21(7.6)	28(7.8)	30(7.9)	22(5.5)	26(6.0)	22(5.4)	33(7.4)	16(4.1)	213(6.6)
Other diseases	6(3.9)	20(7.2)	55(15.3)	69(18.1)	56(14.0)	64(14.7)	56(13.7)	46(10.4)	53(13.7)	425(13.1)

**Table 2:** Spatiotemporal clusters of tuberculosis cases in the study areas, 2010–2018.

Clusters	Longitude (E)	Latitude (N)	Radius (km)	Time Frame	Population	No. Counties	Annual Cases/100,000	Observed/Expected	LLR	RR
1	116.37	28.95	43.74	3/1/2015–10/31/2017	185,214	10	66.3	2.45	106.02	2.61
2	115.84	28.67	0	6/1/2011–9/30/2013	183,246	1	6.8	0.25	47.85	0.24
3	115.86	28.77	0.74	8/1/2011–2/28/2014	831,09	2	2.3	0.086	41.24	0.085
4	115.88	28.72	3.04	1/1/2010–8/31/2012	148,000	2	8.1	0.3	37.12	0.29
5	115.68	28.39	14.57	2/1/2015–9/30/2017	166,248	4	49.4	1.83	34.5	1.89
6	115.81	28.68	0	6/1/2015–12/31/2017	21,862	1	90.2	3.33	25.89	3.37
7	115.83	28.7	2.43	1/1/2010–5/31/2010	144,047	2	0	0	16.15	0
8	115.66	28.58	4.02	9/1/2016–11/30/2018	81,779	3	51.1	1.89	15.88	1.92
9	115.81	28.62	5.03	1/1/2010–7/31/2012	76,100	2	9.7	0.36	14.8	0.35

LLR: Log likelihood ratio; RR: Relative Risk.

## Data Availability

The dataset analyzed is available from the corresponding author for reasonable requests.
